# Preparation of Collodion

**Published:** 1849

**Authors:** 


### ARTICLE IV.

*Preparation of Collodion, or Solution of Gun-cotton as an Adhesive Material for Surgical Purposes.*—(Annalist.)

M. Malgaigne has recently communicated to the French medical journals some remarks on the preparation of gun-cotton for surgical purposes Several French chemists, at the suggestion of M. Malgaigne, attempted to make an ethereal solution of this compound by pursuing the process recommended by Mr. Maynard, in the American Journal of Medical Sciences; but they failed in procuring the cotton in a state in which it could be dissolved by ether. It appears that these experimentalists had employed a mixture of nitric and sulphuric acids; but M. Mialhe ascertained after many trials, that the collodion, in a state fitted for solution, was much more easily procured by using a mixture of nitrate of potash and sulphuric acid.

For the information of our readers who may be disposed to try this new adhesive material, we here give a description of M. Mialhe's process for its preparation. It appears from the results obtained by this chemist, that cotton, in its most explosive form, is not the best fitted for making the ethereal solution.

| | Parts by weight. |
|---|---|
| Finely powdered nitrate of potash - - - | 40 |
| Concentrated sulphuric acid* - - - - - | 60 |
| Carded cotton - - - - - - - - - - - - | 2 |

Mix the nitre with the sulphuric acid in a porcelain vessel, then add the cotton, and agitate the mass for three minutes by the aid of two glass rods. Wash the cotton without first pressing it, in a large quantity of water, and, when all acidity is removed (indicated by litmus paper), press it firmly in a cloth. Pull it out in a loose mass, and dry it on a stove at a moderate heat.

The compound thus obtained is not pure fulminating cotton: it always contains a small quantity of sulphuric acid, is less inflammable than gun-cotton, and it leaves a carbonaceous residue after explosion. It has, however, in a remarkable

*The common commercial acid will answer. When very weak, a longer immersion of the cotton is required.

degree the property of solubility in ether, especially when mixed with a little alchohol, and it forms therewith a very adhesive solution, to which the name of *Collodion* has been applied,

*Preparation of Collodion.*

| | Parts by weight. |
|---|---|
| Prepared cotton - - - - - - - - - - | 8 |
| Rectified sulphuric ether - - - - - | 135 |
| Rectified alcohol - - - - - - - - - - | 8 |

Put the cotton with the ether into a well stoped bottle, and shake the mixture for some minutes. Then add the alcohol by degrees, and continue to shake until the whole of the liquid acquire a syrupy consistency. It may then be passed through a cloth, the residue strongly pressed, and the liquid kept in a well secured bottle.

*Collodion* thus prepared posesses remarkable adhesive properties. A piece of linen or cotton cloth covered with it, and made to adhere by evaporation to the palm of the hand, will suport, after a few minutes, without giving way, a weight of from twenty to thirty pounds. Its adhesive power is so great, that the cloth will commonly be torn before it gives way. The collodion cannot be regarded as a perfect solution of the cotton. It contains, suspended and floating in it, a quantity of the vegetable fibre which has escaped the solvent action of the ether. The liquid portion may be separated from these fibres by a filter, but it is doubtful whether this is an advantage. In the evaporation of the liquid, these undissolved fibres, by feltering with each other, appear to give a greater degree of tenacity and resistance to the dried mass.

In the preparation of collodion it is indespensible to avoid the presence of water, as this renders it less adhesive; hence the ether, as well as the alcohol, should be pure and rectified. The parts to which the collodion is to be applied should be first thoroughly dried, and no water allowed to come in contact with them until all the ether is evaporated.

